# Clinical Impact of Preoperative Magnetic Resonance Imaging in the Evaluation of Myometrial Infiltration and Lymph-Node Metastases in Stage I Endometrial Cancer

**DOI:** 10.3389/pore.2021.611088

**Published:** 2021-04-01

**Authors:** Dorottya Bús, Gyöngyi Nagy, Róbert Póka, György Vajda

**Affiliations:** ^1^ Department of Obstetrics and Gynecology, Zala County Saint Rafael Hospital, Zalaegerszeg, Hungary; ^2^ Department of Radiology, Zala County Saint Rafael Hospital, Zalaegerszeg, Hungary; ^3^ Clinic of Obstetrics and Gynecology, University of Debrecen, Debrecen, Hungary; ^4^ Faculty of Health Sciences, University of Pécs, Pecs, Hungary

**Keywords:** endometrial, MRI, Cancer, Radical hysterectomy, postoperative stage, preoperative stage

## Abstract

**Abstract:** Purpose: In the developed world, endometrial cancer is one of the most common malignant gynecological cancer types. Due to the highly available diagnostic modalities and patient education, the early detection of the tumor leads to high overall survival.

**Methods:** In this study we analyzed the reliability of preoperative MRI findings in the staging of early stage endometrial cancer, as well as the clinical characteristics of patients underwent radical hysterectomy and the histopathologic evaluation of their tumor, with the retrospective data of radical hysterectomies performed in our hospital between 2010 and 2019.

**Results:** The accuracy, sensitivity, specificity, negative- and positive predictive value of MRI regarding stage were 94.7, 63.3, 94.8, 83.8, and 83.8%, respectively. The accuracy, sensitivity, specificity, negative- and positive predictive value of MRI for the detection of the myometrial invasion were 69.8, 80.0, 60.8, 64.3, and 77.5%, respectively. The accuracy, sensitivity, specificity, negative- and positive predictive value of MRI for the detection of lymph node metastases were 78.1, 28.6, 82, 11.1, and 93.6%, respectively.

**Conclusions:** Based on our results, MRI is the method of choice in terms of evaluating overall staging, as well as myometrial invasion, as its specificity and negative predictive value are relatively high. However, systematic lymphadenectomy showed improved cancer-related survival and recurrence-free survival. Our studies showed that the diagnosis of lymph node metastases is difficult with MRI modality since hyperplastic and metastatic nodes cannot easily differentiate, leading to a high percentage of false-positive results. Therefore, other imaging modalities may be used for more accurate evaluation. New findings of our study were that the role of the radiologist’s expertise in the evaluation of MR imaging plays an essential role in lowering false-negative and false-positive results. Therefore, findings evaluated by a radiologist with high-level expertise in gynecological imaging can complement the clinical findings and help substantially define the needed treatment.

## Introduction

Endometrial cancer is the ninth most common tumor among women in developing countries. According to the National *Cancer* Institute Surveillance, Epidemiology, and End Results (SEER) Program, the International Agency for Research on *Cancer* (IARC) [1], and the Hungarian National *Cancer* databases [2], the incidence and mortality of uterine cancer is increasing. However, the 5-years relative survival rate is still around 81.2% overall, with a 95% survival rate in localized tumors—based on SEER 18 data of 2009–2016 [3].

Most endometrial cancer cases are diagnosed in women aged between 45 and 74.67.2% of the tumors are diagnosed at an early stage. Risk factors include extended hyperestrogenism, obesity, diabetes, and hypertension. Symptoms of the cancer are postmenopausal bleeding and premenopausal menstrual disorder [4].

Diagnosis is based on dilatation and curettage, minimally invasive methods (endometrial biopsy, hysteroscopy), and abnormally thickened endometrium on transvaginal ultrasound scan [5]. Magnetic resonance (MR) imaging is the modality of choice for staging, with computed tomography (CT) having relatively low specificity, especially for myometrial invasion. With relatively high specificity in terms of evaluating locoregional spreading, MRI can diminish the extension of hysterectomy [6].

Endometrial cancer is generally staged according to the International Federation of Gynecology and Obstetrics (FIGO) and TNM system, based on histopathological characteristics, tumor grade, rate of myometrial invasion, the presence or absence of lymph-node and distant metastases [7, 8]. As standard treatment of early stage endometrial cancer is total abdominal hysterectomy and adnexectomy, preoperative staging is crucial to further determine the radicality of surgery and the required neoadjuvant and adjuvant oncologic treatment. Pelvic or pelvic-paraaortic lymphadenectomy performed in early stages is still controversial and discussed in international literature [9].

Our study aimed to examine the accuracy of magnetic resonance imaging in TNM T1 stage cancers in evaluation of myometrial invasion and lymph node metastases compared with final histopathologic results.

## Material and Methods

Our results were based on a retrospective, one-institute dataset between the years 2010 and 2019.

In this study, we examined the reliability of magnetic resonance imaging in stage I uterine endometrial adenocarcinoma cases of 9 years, selecting the patients with tumor of TNM stage 1 (TNM T1a or T1b), with or without lymph-node metastases (TNM N0 or N1), based on the final histopathologic evaluation of the surgical specimens. Patients with cervical, vaginal, parametrial, or locoregional involvement (TNM T2-4) or patients with distant metastases (TNM M1) were excluded from the study.

Between 2010 and 2019, 148 radical hysterectomies and lymphadenectomies were performed at the Department of Obstetrics and Gynecology of Zala County Saint Rafael Hospital due to endometrial adenocarcinoma of the uterus. 64.9% of the cases, a total of 96 surgical specimens were evaluated as TNM stage 1 tumors.

The following data were obtained from the Medical Network System database of the hospital: Age of the patient at the time of surgery, preoperative MRI staging, surgical description of the radical hysterectomy and lymphadenectomy, histopathological type of the tumor, postoperative treatment (irradiation, chemotherapy), recurrence of tumor or metastases in the follow-up period (from surgery until December 2019), 1- and 5-years mortality.

On all patients with abnormal vaginal bleeding, dilatation and curettage were performed. Based on a positive histopathological result of endometrial cancer, a preoperative radiological investigation was performed for local and distant cancer staging. Modalities involved magnetic resonance imaging, chest X-ray, transvaginal ultrasound and computed tomography, when necessary.

**FIGURE 1:**
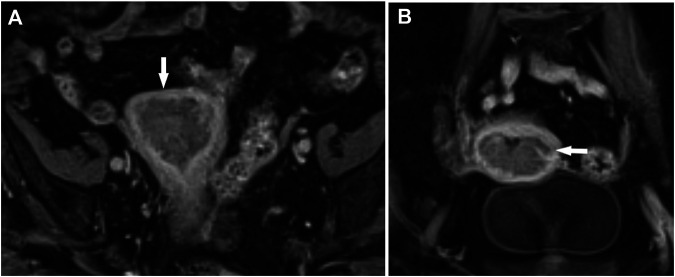
Endometrial cancer, stage T1b. Myometrial infiltration >50%. Post-contrast dynamic fat-sat T1 weighted VIBE image (fat-suppressed Volumetric interpolated breath-hold examination), **(A)** coronal view, **(B)** axial view.

**FIGURE 2:**
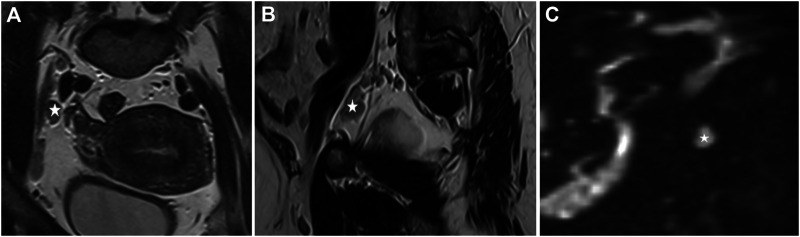
Endometrial cancer, stage T1b. *: Enlarged lymph nodes **(A)**. T2 sequence, coronal view, enlarged lymph nodes near the external iliac artery, **(B)** T2 sequence, sagittal view, an enlarged lymph node near the external iliac artery, **(C)** enlarged lymph node, postcontrast DWI (Diffusion-weighted magnetic resonance imaging, b:800).

The preoperative MRI was performed using a Siemens Magnetom Area 1.5 T device. Data required for FIGO 2009 [7] and TNM [8] staging were collected: the degree of myometrial, serosal, adnexal, and parametrial invasion, as well as metastases of pelvic or para-aortic lymph nodes. Measurement of the deepest myometrial tumor invasion was done and classified into stage FIGO IA/TNM T1a (invasion depth less than half of the myometrial thickness) or FIGO stage IB/TNM 1 B tumor (invasion depth more than half of the myometrial thickness). Pelvic or para-aortic lymph node metastases were suspected based on nodal size criterion (short axis >1 cm). Images of the retroperitoneal, parailiac, pelvic, and cervical area were taken, with gadolinium-based contrast medium, sequences seen in Table 1 (Figures 1, 2).

**TABLE 1:** Imaging protocol of MRI.

Precontrast sequences						
Sequence	Voxel size (mm)	FoV (mm)	Slice thickness (mm)	Slice number	TR (ms)	Te (ms)
Coronal TRUFISP T2	1.0 × 1.0 × 5.0	330	5.0	26	4.17	1.62
Coronal T1 spin echo	0.9 × 0.9 × 4.0	400	4.0	26	589	12
Sagittal T2 spin echo	0.5 × 0.5 × 3.5	240	3.5	48	3,930	97
Axial T2 spin echo	0.5 × 0.5 × 4.0	240	3.5	36	3,080	93
Coronal T2 spin echo	0.5 × 0.5 × 3.5	240	3.5	36	3,080	93
Axial and sagittal DWI	2.0 × 2.0 × 5.5	380	5.5	20	4,700	85
Axial fat-sat T1 spinecho	1.2 × 1.2 × 4.5	300	4.5	32	584	7.8
Axial and coronal fat-sat T1 VIBE (until 2019 January)	1.4 × 1.4 × 1.4	400	1.4	Slices per slab: 144	6.82	2.89
**Postcontrast sequences**						
** Sequence**	**Voxel size (mm)**	**FoV (mm)**	**Slice thickness (mm)**	**Slice number**	**TR (ms)**	**TE (ms)**
Axial and sagittal fat-sat T1 spinecho	1.2 × 1.2 × 4.5	300	4.5	28	621	7.8
Coronal fat-sat T1 VIBE (until 2019 January)	1.4 × 1.4 × 1.4	400	1.4	Slices per slab: 144	7.08	2.39
Dynamic fat-sat T1 VIBE (until 2019 January)	1.4 × 1.4 × 1.8	400	1.8	Slices per slab: 104	7.08	2.39

Postcontrast sequences with gadolinium-based contrast medium (TRUFISP, true, fast imaging with steady-state free precession; DWI, Diffusion-weighted magnetic resonance imaging; Fat-sat, Fat suppression; VIBE, Volumetric interpolated breath-hold examination).

Based on radiologic staging, a multidisciplinary tumor board, consisting of an oncologist, a pathologist, a radiologist, and a gynecologist, with a distant radiotherapist consultant, if needed, decided on the necessity of neoadjuvant oncological therapy and the radicality of hysterectomy with the possibility of involving a multidisciplinary surgical team was considered in case of local intestinal or urological metastasis.

Abdominal hysterectomy and adnexectomy was in all cases performed by a gynecologist and an urologist specialist trained in oncosurgery, combined with lateral extension in high-risk cases according to the ESMO-ESGO-ESTRO (2010 and 2016 revision) guidelines [9, 10].

**FIGURE 3:**
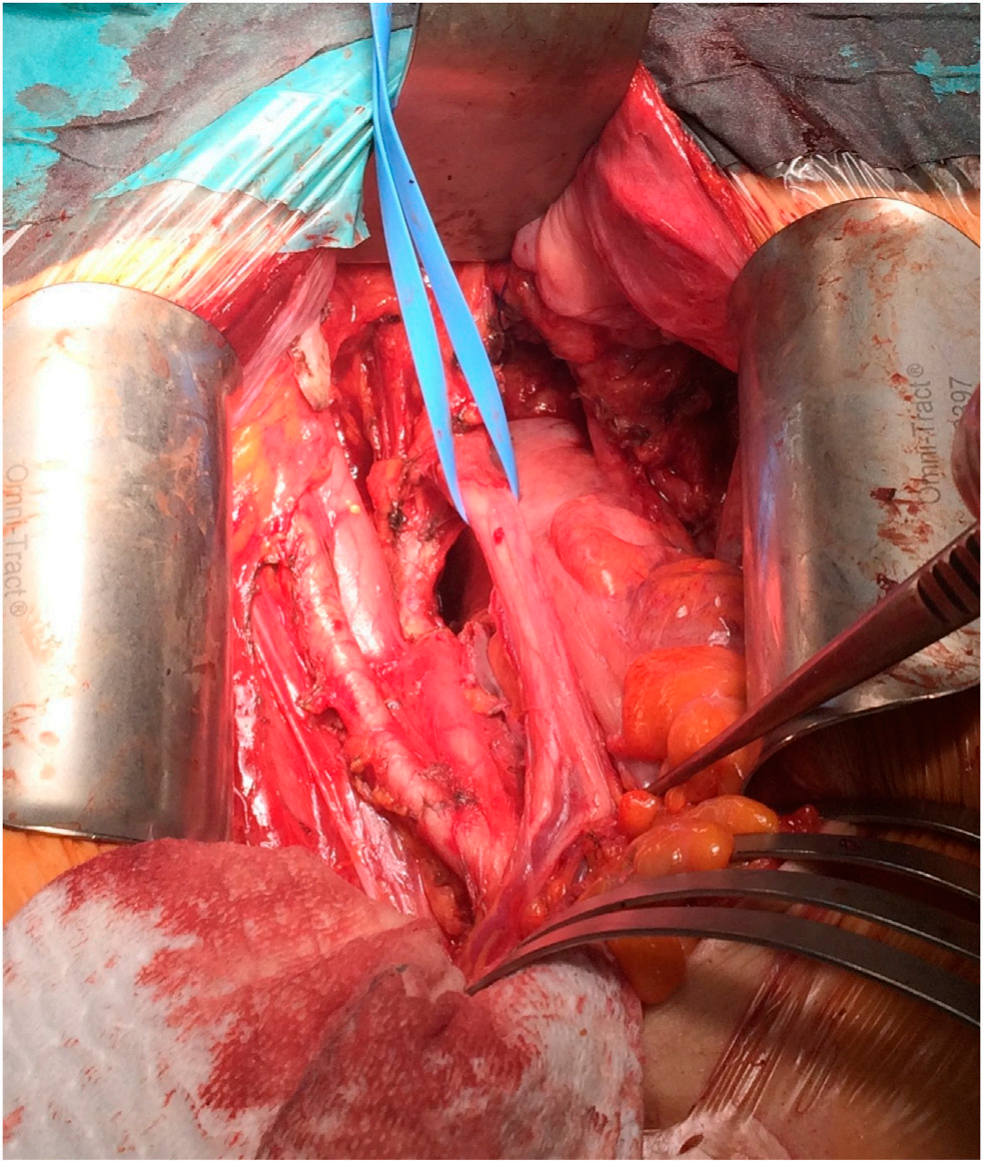
Intraoperative picture, with the preparation of parailiacal and obturator space.

Radical hysterectomy involved removing the cervix, uterus, Fallopian tubes, and ovaries, together with the parametria and upper vagina, followed by parailiacal and pelvic/obturator lymphadenectomy in the pelvic retroperitoneal space (Figure 3).

Macroscopic and microscopic histopathological evaluation of the surgical specimen was performed. Findings included the description of the type of tumor, differentiation, TNM, and FIGO staging based on the extent of myometrial infiltration (<50%, ≥50%), and the presence or absence of parametrial, cervical, adnexal, rectal, and cystic involvement, lymph-node, and pelvic wall invasion.

The staging was re-evaluated by the tumor board postoperatively, based on intraoperative and histopathological findings. A postoperative oncological therapy was designed individually for each patient, based on the ESMO Guidelines. Patients with stage 1, low-grade endometrioid endometrial cancer received adjuvant vaginal brachytherapy; high-grade cases were referred to external beam radiation therapy (EBRT). Patients with positive lymph node status received adjuvant systemic, platinum-based chemotherapy, and radiotherapy (EBRT or vaginal brachytherapy) [9, 10].

Conducting follow-up visits in the Department of Oncology and the Department of Gynecology included routine physical and radiological check-ups. The follow-up frequency was six weeks, three months, six months, one year, then annually after surgery. Follow-up data were obtained from surgery until December 2019.

Data collection was approved by the Ethics Committee of the Hospital, according to the Declaration of Helsinki.

## Statistical Analysis

The age at the time of surgery, the prevalence of different tumor histological subtypes, tumor grades, and differentiation were taken into account to analyze tumors of the final histopathologic staging as stage I endometrial cancer. The 1- and 5-years survival rates were calculated, accounting for deaths occurring before December 2019 (Table 2).

**TABLE 2:** Patient and tumor characteristics.

Age (year)	63.52 ± 9.4 (37–84)	Grade	
Gravidity	2.28 ± 1.47 (0–10)	Low	26 (27.1%)
Parity	1.69 ± 0.93 (0–4)	High	70 (72.9%)
Postmenopause	91 (94.8%)	Treatment	
Histologic subtype					
Endometrioid adenocarcinoma	90 (93.7%)	Adjuvant irradiation	87 (90.6%)
Serous adenocarcinoma	6 (6.3%)	Adjuvant chemoirradiation	4 (4.1%)
		Adjuvant chemotherapy (due to recurrence or metastases)	3 (3.1%)
Positive lymph node status	26 (27.1%)	Recurrence and/or metastasis	8 (8.3%)

Age, gravidity, parity: mean, SD, range. Postmenopausal state, Histopathologic subtype, Grade, Treatment, Recurrence, and metastasis: frequency (%).

The preoperative radiology staging results were compared with the final histological analysis of the surgical specimen, using χ2 or Fisher’s exact test to compare variables. *p*-values < 0.05 were considered to be significant. The sensitivity, specificity, positive- and negative predictive values of the preoperative assessment were calculated for each endpoint, together with 95% confidence intervals (95% CI). The percentage of the underdiagnosed or overdiagnosed cases and accuracy rate in terms of stage, myometrial invasion, and the number of lymph node metastases were also calculated.

All data were collected in an Excel database, and they were analyzed using SPSS statistical software. The intraclass correlation coefficient (ICC) was used to determine the inter-rater agreement between the radiologist and the pathologist concerning overall staging, myometrial invasion, and lymph node metastases. ICC below 0.50 was considered poor, between 0.50 and 0.75 as moderate, between 0.75 and 0.90 as good, and above 0.90 ICC, it was considered excellent inter-rater reliability. SPSS software (ver. 25; SPSS Inc., Chicago, IL, United States) was used for the statistical analyses, and *p*-values < 0.05 were considered significant.

## Results

Our results were based on a retrospective, one-institute dataset between the years 2010 and 2019. We analyzed the data of 96 patients with the final histopathologic evaluation of TNM T1 stage endometrial adenocarcinoma tumors, with the follow-up period from their surgery until December 2019. One patient with endometrial sarcoma was excluded from the study.

The patients’ age with the final histopathologic result of TNM staged T1 endometrial cancer was from 37 to 84 years with a mean of 63.52±9.4 years. Most of the patients were postmenopausal (94.8%) and overweight (body mass index >25 kg/m2; 76.4%).

According to the tumor board’s decision, 87 patients (90.6%) received adjuvant irradiation, and four of them (4.1%) had the treatment of chemoradiation due to local recurrence and distal metastases occurring in the follow-up period. In the follow-up period, five of the patients (5.2%) were diagnosed with metastases (lung, osseal, and lymph-node), and local recurrence occurred in three of the patients (3.1%); three of these patients received chemotherapy.

In the follow-up period, overall, eight patients died; four of the deaths were endometrial-cancer related. The overall 1-year mortality rate was 2.1% (1% cancer-related); the 5-years mortality rate was 8.3% (4.2% cancer-related).

Postoperative histologic assessment revealed histopathologic subtype of endometrioid adenocarcinoma in 92.8%, serous adenocarcinoma in 6.2%, and sarcoma in 1% of the cases. The patient with endometrial sarcoma was excluded from the study. 72.9% of the cases were diagnosed as high-grade, 27.1% as low-grade tumors (Table 2).

In stage I cancers, the accuracy of MRI regarding stage was 94.7%, and its sensitivity, specificity, PPV, and NPV were 63.3, 94.8, 83.8, and 83.8%, respectively, with the intraclass correlation coefficient of 0.782 (0.697–0.842) (with 95% confidence interval [CI]; *p* < 0.001). Rates of underdiagnosis were 5.2% of the cases (Tables 3, 4). Based on the results, MRI is highly accurate and specific in overall staging, with good inter-rater reliability compared to final histopathologic findings.

**TABLE 3:** Results. Accuracy, sensitivity, specificity, PPV, and NPV regarding TNM staging, myometrial invasion, lymph node metastases, and MRI results were compared with final histopathologic evaluation.

	Overall*	Myometrial invasion*	Lymph-node metastases
Accuracy	94.7%	69.8%	78.1%
Sensitivity	63.3%	80.0%	28.6%
Specificity	94.8%	60.8%	82%
Positive predictive value	83.8%	64.3%	11.1%
Negative predictive value	83.8%	77.5%	93.6%
Intraclass correlation coefficient	0.782	0.576	0.117

Intraclass correlation coefficient (ICC): <0.50—poor, 0.50–0.75—moderate, 0.75–0.90—good inter-rater reliability (CI 95%) *p < 0.05

The accuracy of MRI for the detection of myometrial invasion in stage I cancers was 69.8%, and its sensitivity, specificity, PPV, and NPV were 80.0, 60.8, 64.3, and 77.5%, respectively, with the intraclass correlation coefficient of 0.576 (0.368–0.716) (with 95% confidence interval [CI]; *p* < 0.001). The rates of overdiagnosis were 20.8%, underdiagnosis was 9.4% (Table 4). Based on the results, MRI is sensitive in terms of myometrial invasion depth, with high negative predictive value and moderate accuracy, specificity, and positive predictive value. Inter-rater reliability moderate in comparison to final histopathologic findings.

**TABLE 4:** Difference between MRI and histology results, myometrial invasion.

	MR imaging		Histopathologic assessment	
	N	%	N	%
T1A (<50% myometrial invasion)	40	41.7	51	53.1
T1B (>50% myometrial invasion)	56	58.3	45	46.9

Mean, surgically removed pelvic lymph node count was 10.4 (SD ± 4.1). The accuracy of MRI for the detection of lymph-node in stage I cases was 78.1%, and its sensitivity, specificity, PPV, and NPV were 28.6, 82, 11.1, and 93.6%, respectively, with the intraclass correlation coefficient of 0.117 (−0.394–0.401) (with 95% confidence interval [CI]; *p* = 0.026). False-negative results were 5.2%, false-positive were 16.7% of the cases (Table 3,). Based on the results, MRI is moderately accurate and specific in local lymph-node staging, with a high negative predictive value. Results showed poor inter-rater reliability in comparison to final histopathologic findings.

To analyze the initial learning curve’s role in evaluating MR images, we compared the results of two rater-group in terms of accuracy, down- and upstaging in regards to myometrial invasion and lymph node spread. A radiologist with high expertize in gynecologic radiology evaluated 60 of the cases, three radiologists with medium-level expertize evaluated 36 cases. A radiologist with higher expertize showed more constant findings and lower upstaging-rate in terms of myometrial invasion than radiologists with no specialization in gynecologic oncology. However, the evaluation of lymph-node metastases was more accurate in the second group, with only four upstaged cases (Table 5).

**TABLE 5:** Role of expertize in the evaluation of MR images.

Myometrial invasion						
	Accuracy		Upstaging		Downstaging	
	N	%	N	%	N	%
Rater 1 (N = 60)	45	75	7	11.7	8	13.3
Rater 2 (N = 36)	23	63.9	12	33.3	1	2.8

Lymph node spread						
	Accuracy		Upstaging		Downstaging	
	N	%	N	%	N	%
Rater 1 (N = 60)	43	71.7	14	23.3	3	5
Rater 2 (N = 36)	32	88.9	4	11.1	0	0

A radiologist with high gynecological radiology expertize evaluated 60 of the cases, three radiologists with medium-level expertize evaluated 36 of the cases.

## Discussion

In the developed world, endometrial cancer is one of the most common malignant gynecological tumor types. Due to the highly available diagnostic modalities and patient education, the early detection of the tumor leads to high overall survival. Prognostic factors include histopathologic subtypes, grade, myometrial invasion, and lymph node metastases [11].

In preoperative staging, MRI is considered the best choice to assess myometrial invasion depth, cervical involvement, and tumor grade. However, its role in the evaluation of lymph node metastases is still controversial [12]. The National Comprehensive *Cancer* Network (NCCN) and the European Society of Urogenital Radiology (ESUR) advise MRI imaging in endometrial cancers with serous histologic subtype, suspected cervical invasions, and identify patients with stage Ia disease. As patients with endometrioid endometrial cancer with myometrial invasion of more than 50%, or with serous-type endometrial cancer are considered to be intermediate to high risk of lymph node metastasis, preoperative staging is essential for a tailored pre-and postoperative treatment and for planning the radicality of the surgical treatment [13].

In this study, we analyzed the reliability of preoperative MRI findings in the staging of early stage endometrial cancer and patients’ clinical characteristics who underwent radical hysterectomy and the histopathologic evaluation of their tumor.

The results of the preoperative radiology staging were compared with the final histological analysis of the surgical specimen. The sensitivity, specificity, positive- and negative predictive values of the preoperative assessment were calculated for each endpoint. The percentage of the underdiagnosed or overdiagnosed cases and accuracy rate in terms of stage, myometrial invasion, and lymph node metastases were also evaluated. Inter-rater agreement was calculated to determine the conformity of pre-and postoperative staging.

According to our findings, the overall accuracy of MRI in regards to staging was 94.7% with high specificity, high positive- and negative predictive value, and low sensitivity, with good inter-rater reliability. As most of the tumors are detected at an early stage, the high sensitivity of MRI for the myometrial invasion in stage I diseases means that this modality plays an essential role in planning the radicality of hysterectomy in localized tumors. Based on the decision of the hospital’s oncologic team, pelvic lymphadenectomy was carried out in most of our cases for accurate surgical staging; however, due to low sensitivity and low positive predictive value of MR imaging for lymph node detection, a considerable number, 16.7%, of surgeries at an early stage were complemented with lymphadenectomy with false-positive MRI results.

In literature reviews of the past few years’ studies, similar results were reported, showing sufficient specificity but low sensitivity regarding lymph node detection due to similar radiological findings of hyperplastic and metastatic lymph-node enlargement (Table 6). [14] Our MRI results in terms of myometrial invasion showed average accuracy, sensitivity, and negative predictive compared to studies; however, the positive predictive value was lower. In terms of lymph-node metastases, sensitivity, and positive predictive value of our results were relatively low; even the radiologist with high expertize upgraded 23.3% of the lymph nodes.

**TABLE 6:** Review of the literature.

Myometrial invasion							
Author	Year	Patient number	Accuracy	Sensitivity	Specificity	Positive predictive value	Negative predictive value
Goel et al. [22]	2018	58	74.14%	75%	73.08%	77.2%	70.37%
Tanase et al. [23]	2018	84	88.1%	82.1%	93.5%		
Yu-ting huang et al. [24]	2019	Review of literature	77–90%	85–94%	60–73%		
Shatat et al. [25]	2019	29	75.86–93.1%	66.7–94.7%	60–94.7%	60–94.7%	66.7–94.7%
Yildirim et al. [26]	2018	40	75%	77.8%	72.7%	70%	80%
Gil et al. [27]	2019	44	61–95%	58–96%	58–96%	55–96%	55–95%
Current study	2020	96	69.8%	80%	60.8%	64.3%	77.5%
**Lymph node spread**							
**Author**	**Year**	**Patient number**	**Accuracy**	**Sensitivity**	**Specificity**	**Positive predictive value**	**Negative predictive value**
Goel et al. [22]	2018	58	86%	88.64%	66.67%	95.12%	44.4%
Tanase et al. [23]	2018	84		74.4%	82.1%		
Yu-ting huang et al. [24]	2019	Review of literature	77–99%	50–85%	90–99%		
Current study	2020	96	78.1%	28.6%	82%	11.1%	93.6%

Accuracy, sensitivity, specificity, PPV, and NPV of MR imaging regarding Myometrial invasion and lymph node spread.

Based on our results, MRI is the method of choice in evaluating overall staging and myometrial invasion, as its specificity and negative predictive value are relatively high [15, 16]. The role of lymph-node status is essential in prognosis and guiding adjuvant treatment [17, 18]. Therefore it should be considered based on the preoperative imaging results [19]. In the retrospective ASTEC study, there was no significant better overall survival or recurrence-free survival when pelvic lymphadenectomy was carried out [20]. However, in the large, retrospective SEPAL study, patients with high-risk histology (low-grade endometrial cancer with myometrial invasion ≥50% or high-grade histology) showed better cancer-related survival, recurrence-free survival with standard surgery with lymphadenectomy than with standard surgery with adjuvant radiotherapy [21]. Our results showed similar findings to the SEPAL study as the survival-rate was high with pelvic lymphadenectomy; therefore, systematic lymphadenectomy can be considered in high-risk cases.

Based on our studies, the diagnosis of lymph node metastases is difficult with MRI modality since hyperplastic and metastatic nodes cannot easily differentiate, leading to a high percentage of false-positive results. Therefore, other imaging modalities, such as ultrasound, CT, and PET-CT [10], can be used for more accurate evaluation; however, due to the lack of sufficient data, we did not investigate these methods’ accuracy.

Limitations to these findings are that the number of removed lymph-nodes and thoroughness of the histopathological evaluations can result in false-negative results; however, hyperplastic lymph-nodes can mimic metastases; therefore, the gold-standard size-criterion of positive evaluation should be revised and used cautiously.

## Conclusion

Based on our results, MRI is considered the best choice to assess myometrial invasion depth, cervical involvement, and tumor grade in preoperative staging. Its role in the evaluation of lymph node metastases is still controversial.

Our study’s strength was that our results and examination data were based on retrospective, one-institution data. The limitation of our findings was that the number of patients was relatively low.

New findings of our study were that the role of the radiologist’s expertize in the evaluation of MR imaging plays an essential role in lowering false-negative and false-positive results; therefore, findings evaluated by a radiologist with high-level expertize in gynecological imaging can complement the clinical findings and help substantially define the needed treatment.

## Data Availability

The raw data supporting the conclusions of this article will be made available by the authors, without undue reservation.
